# The endopeptidase of the maize-affecting *Marafivirus* type member maize rayado fino virus doubles as a deubiquitinase

**DOI:** 10.1016/j.jbc.2021.100957

**Published:** 2021-07-12

**Authors:** Ankoor Patel, Jessica A.M. McBride, Brian L. Mark

**Affiliations:** Department of Microbiology, University of Manitoba, Winnipeg, Canada

**Keywords:** maize rayado fino virus, MRFV, *Tymoviridae*, papain-like protease, ubiquitin, deubiquitinase, ubiquitin hydrolase, polyprotein, RNA viruses, structural biology, +ssRNA, positive-sense ssRNA, AMC, 7-amino-4-methylcoumarin, ASU, asymmetric unit, BlVS, blackberry virus S, CP, coat protein, CSDaV, citrus sudden death–associated virus, CSS, complex formation significance score, DUB, deubiquitinase, GSyV1, Grapevine Syrah virus 1, HEL, helicase, MR, molecular replacement, MRFV, maize rayado fino virus, OBDV, oat blue dwarf virus, OLV3, Olive latent virus 3, OTUD3, ovarian tumor domain–containing protein 3, PROs, proteases, RdRp, RNA-dependent RNA polymerase, TYMV, turnip yellow mosaic virus, TYMV PRO, papain-like cysteine protease of TYMV, Ub, ubiquitin, Ub-3Br, ubiquitin(1–75)–3-bromopropylamine

## Abstract

*Marafiviruses* are capable of persistent infection in a range of plants that have importance to the agriculture and biofuel industries. Although the genomes of a few of these viruses have been studied in-depth, the composition and processing of the polyproteins produced from their main ORFs have not. The *Marafivirus* polyprotein consists of essential proteins that form the viral replicase, as well as structural proteins for virus assembly. It has been proposed that *Marafiviruses* code for cysteine proteases within their polyproteins, which act as endopeptidases to autocatalytically cleave the polyprotein into functional domains. Furthermore, it has also been suggested that *Marafivirus* endopeptidases may have deubiquitinating activity, which has been shown to enhance viral replication by downregulating viral protein degradation by the ubiquitin (Ub) proteasomal pathway as well as tampering with cell signaling associated with innate antiviral responses in other positive-sense ssRNA viruses. Here, we provide the first evidence of cysteine proteases from six different *Marafiviruses* that harbor deubiquitinating activity and reveal intragenus differences toward Ub linkage types. We also examine the structural basis of the endopeptidase/deubiquitinase from the *Marafivirus* type member, maize rayado fino virus. Structures of the enzyme alone and bound to Ub reveal marked structural rearrangements that occur upon binding of Ub and provide insights into substrate specificity and differences that set it apart from other viral cysteine proteases.

Positive-sense ssRNA (+ssRNA) viruses have evolved remarkable polycistronic translational mechanisms that maximize genomic coding capacity to produce the viral proteins needed for replication and packaging (1, 2). These include programmed ribosomal frameshifting, polyprotein expression and processing, and the presence of subgenomic mRNAs (2, 3). Unlike the highly divergent DNA viruses that typically use alternative splicing of viral mRNA to produce their full arsenal of proteins (4, 5), +ssRNA viruses most commonly have their nonstructural genes translated directly from their genomes *via* large ORFs (3, 5). Upon translation, these ORFs give rise to precursor polyproteins that can be processed into functional units by endopeptidases found within the polyprotein or by cellular proteases that achieve the same purpose (5). This expression mechanism allows viruses to produce a full set of proteins from their genomes without needing to encode for additional genetic features that would typically direct and regulate translation. +ssRNA viruses infect virtually all forms of life (6). Significant attention has been paid to human- and animal-affecting +ssRNA viruses that are capable of cross-species transmission; however, the +ssRNA virome affecting plants appears to be far more diverse and abundant (6, 7). With the threat of food shortage becoming more of a reality in a matter of decades because of climate change and the quickly increasing global population (8, 9), understanding the relationship between crops important to global food security and their viral pathogens will aid in developing sustainable agricultural practices.

*Marafiviruses* are +ssRNA viruses, which cause persistent infection in diverse plant species that are agriculturally relevant to food and biofuels production (10, 11, 12). Currently, there are ten classified members of the genus *Marafivirus* (13), and additional *Marafivirus* candidates that affect different plant species have been identified recently (14, 15). *Marafiviruses* are persistent propagative viruses, meaning that they are also able to replicate in the leafhopper insect vectors that transmit them to their final plant host (16). *Marafiviruses* have a single-stranded genome with an average size of 6 to 7 kb, which is translated into a single polyprotein that is roughly 2000 amino acids in length (Fig. 1*A*). The polyprotein characteristically contains four nonstructural domains that comprise the viral replicase (methyltransferase, protease (PRO), helicase (HEL), and RNA-dependent RNA polymerase (RdRp)) as well as the major structural coat protein (CP) for genome packing (17). Maize rayado fino virus (MRFV) is the type member of the genus *Marafivirus* within the family Tymoviridae (10). The virus was first described in 1969 from Costa Rica and has since been found as far south as Brazil and as far north as the United States (10, 18, 19). MRFV infection causes reduction in the height and ear development with few to no seeds within certain corn species and can lead to severe agricultural loss with decimation to nearly 100% in some cultivars (20).

**Figure 1:**
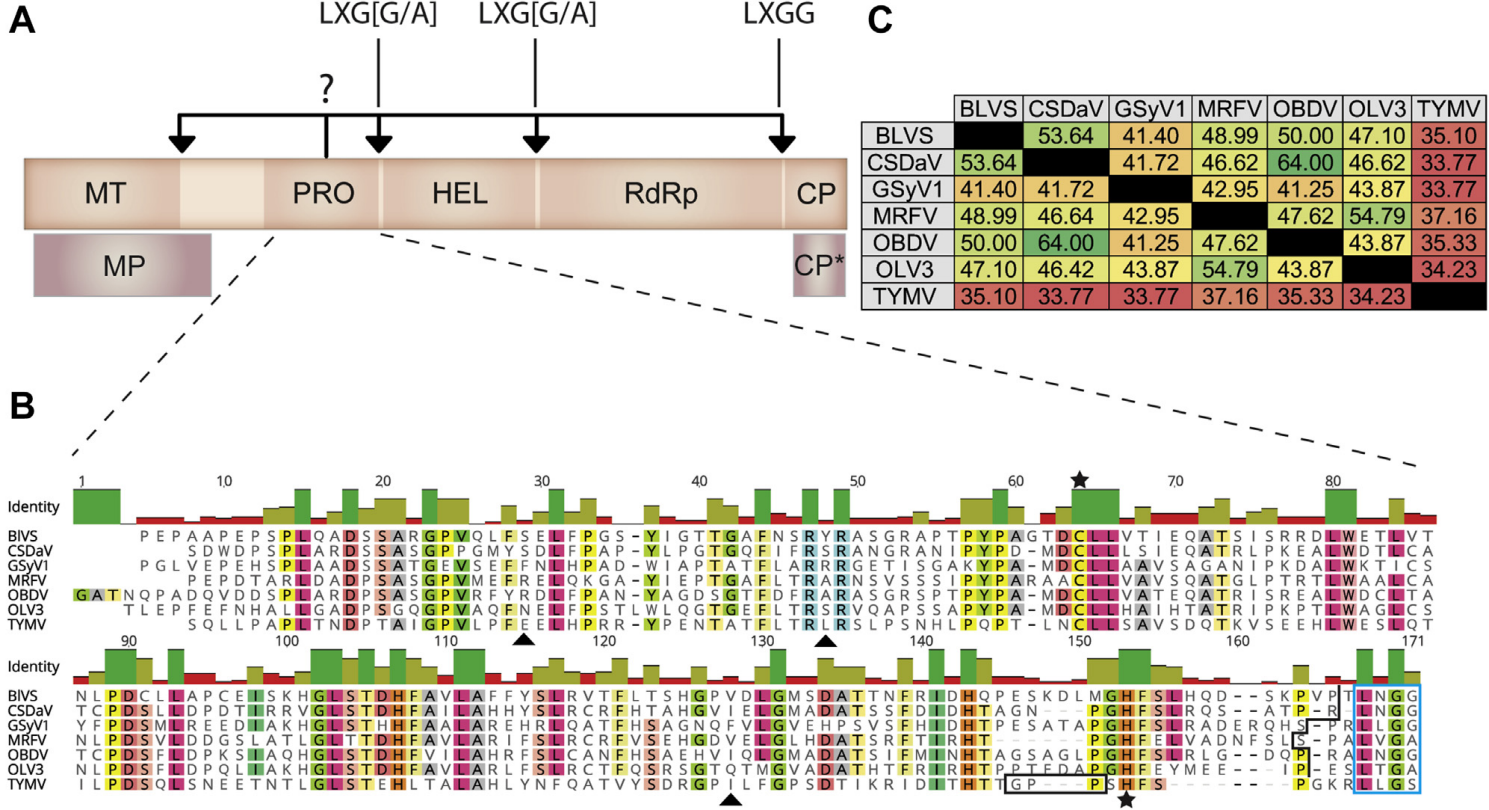
***Marafivirus* polyprotein arrangement and endopeptidase comparison.***A*, simplified schematic of the typical arrangement of the sole polyprotein produced by *Marafiviruses* and two additional proteins produced from genomic RNA. *Arrows* indicate junction points between each domain and the possible cleavage sites targeted by the endopeptidase. The four amino acid residues at each putative cleavage sites are indicated where “X” is any amino acid. *B*, sequence alignment of PRO domains from six *Marafiviruses* and one *Tymovirus*. *Highlighted* residues indicate agreement of ≥65%. *Stars* indicate the catalytic cysteine and histidine of the active sites, and the *triangle* indicates a potential triad residue. *Triangles* indicate key residues. The *box* around the C terminus GPP of TYMV is the mobile loop characteristic to *Tymoviruses*. The *stepwise line* at the C terminus of the *Marafivirus* PRO domains indicate where the expression constructs used herein were terminated. Alignment was done in Geneious v.11.1.5. *C*, percent identity matrix of all six *Marafivirus* PRO domains and the PRO domain of TYMV. Values are presented in the heat map format, where *green* indicates a higher degree of sequence similarity. Multiple sequence align matrix values were determined using Clustal Omega (70). BlVS, blackberry virus S; CP, major coat protein; CP∗, minor coat protein; CSDaV, citrus sudden death–associated virus; GSyV1, Grapevine Syrah virus 1; HEL, helicase; MP, movement protein; MRFV, maize rayado fino virus; MT, methyl transferase; OBDV, oat blue dwarf virus; OLV3, Olive latent virus 3; PRO, protease; RdRp, RNA-dependent RNA polymerase; TYMV, turnip yellow mosaic virus.

MRFV shares remarkable sequence similarity to the *Tymovirus* type member, turnip yellow mosaic virus (TYMV) (∼43% at the amino acid level); however, the amount of foundational research that exists between the two is disparate, with TYMV being far better studied. The papain-like cysteine protease of TYMV (TYMV PRO) is currently the most extensively characterized plant deubiquitinase (DUB) (21, 22, 23, 24, 25). Over 67% of plant viral proteases, like all the *Marafivirus* PROs, are cysteine proteases (26). The main function of PRO is to act as a polyprotein processor, but it has been shown to have auxiliary function as a ubiquitin (Ub) hydrolase to aid in bypassing the host innate immune system by removing Ub from the RdRp, preventing its degradation by the Ub proteasome (21, 24, 25, 27). Notably, the junctions between the replicase domains within the *Marafivirus* polyprotein (Fig. 1*A*, arrows) closely resemble the C-terminal ^73^LRGG^76^ tail of Ub (the cleavage site for Ub hydrolases) (28), suggesting *Marafivirus* endopeptidases may also have deubiquitinating activity.

Protein ubiquitination is a highly conserved post-translational modification process that occurs in eukaryotes including plants, which regulates the function, trafficking, and fate of protein substrates in the cell (28, 29). Ubiquitination involves the tethering of a Ub molecule(s) to a protein substrate by the combined efforts of Ub-activating enzymes, Ub-conjugating enzymes, and Ub ligases (28, 30). Ub is typically conjugated to the substrate protein as a polyubiquitin chain (28). The first Ub molecule is covalently attached to a lysine on the surface of a substrate protein *via* the carboxy-terminal glycine of Ub, forming a covalent isopeptide bond with the lysine side chain ε-amino group (28, 30). Additional Ub molecules can be attached to the first conjugated Ub through linkages formed between one of seven lysine residues on the surface of Ub (Lys6, Lys11, Lys27, Lys29, Lys33, Lys48, or Lys63) and the terminal Gly of the newly added Ub molecule (29). Met1-linked chains occur in mammalian systems, although they have not been identified in plant cells (31). Typically, Ub chains are of the same linkage type, but mixed poly-Ub chains are known to exist (28). The nature of the conjugation type and/or length ultimately determines the function or fate of the protein substrate (30, 32).

In the model plant organism, *Arabidopsis thaliana*, 6% of all protein-encoding genes are purportedly linked to some facet of Ub modification (33), and 12 genes have been identified to date that code for functional Ub or Ub-like proteins such as RUB proteins (31), illustrating the large role that the Ub system has in plants. Lys48 and Lys63 are the most well-understood poly-Ub chain types and the two most abundant forms in plants (34). Lys48 polyubiquitination (and Lys63 to a lesser extent) marks a substrate protein for degradation in plants by the Ub-proteasome system (30, 35, 36), whereas Lys63 has many different roles in plant cells such as DNA replication/repair, iron homeostasis, endocytosis, nutrient transport, vacuolar sorting, protein synthesis, and immunity (31, 37).

Here, we provide the first structural and functional insight into the endopeptidases from the genus *Marafivirus*. We present data illustrating that the PRO domains from six different species within this genus all have *bona fide* deubiquitinating activity. We compare the differences and similarities of all six PROs toward recognition of different types of Ub substrates and contrast our findings with those of the *Tymoviruses*. We also discuss the endopeptidase activity of MRFV PRO that is required for polyprotein processing to generate the viral replicase complex. X-ray structures of MRFV PRO alone and bound to Ub reveal unique structural characteristics that differ from TYMV PRO and notable conformational changes that occur in response to Ub substrate. Together, our data provide new insight into how *Marafiviruses* generate their replicase machinery through polyprotein processing and that they exhibit DUB activity that may corrupt the immune response of their hosts.

## Results and discussion

### Comparative sequence analysis of the PRO domains of six *Marafiviruses* and one *Tymovirus*

As shown in Figure 1*A*, the majority of the *Marafivirus* proteome exists within one major polyprotein (∼200 kDa) consisting of the core replication proteins needed for the virus to replicate its genome within the host (10, 38). Structural proteins are also present within the polyprotein as well as the sole endopeptidase PRO (38). The methyltransferase, PRO, HEL, RdRp, and CP domains are consistently found in this arrangement within the polyprotein. The PRO domains are believed to be involved with the processing of the polyproteins into individual functional subunits by cleavage of the putative scissile bond(s) as observed for *Tymoviruses* such as TYMV (22, 39, 40, 41).

An amino acid sequence alignment of the six *Marafivirus* PRO domains we examined reveals considerable variability in the percent identity between the *Marafivirus* enzymes and consistently low identity to TYMV PRO although they appear to serve the same purpose (Fig. 1, *B* and *C*). Regardless, the similarity between the *Marafivirus* and TYMV PRO sequences was sufficient to identify *Marafivirus* PRO domains based on alignments against the TYMV PRO sequence for which an X-ray crystal structure has been determined (Fig. 1*B*). The PRO domains from six *Marafiviruses* were explored: BlVS, CSDaV, GSyV1, MRFV, OBDV, and OLV3. The DNA sequence for each PRO was codon-optimized for expression in *E. coli*. Amino acid sequences for the *Marafiviruses* had been derived from RNA sequences deposited in the NCBI (Table S1). Figure 1*B* shows the amino acid sequences of each PRO domain that was expressed. As determined here, functionally active *Marafivirus* PROs are compact with an average sequence length and molecular weight of ∼160 residues and ∼17 kDa, respectively. The *Marafivirus* PRO enzymes we studied share at least 40% sequence identity, with CSDaV and OBDV having a remarkable 64% sequence identity (Fig. 1*C*). Comparing the sole *Tymovirus* PRO (TYMV) with the sequences of the PROs from the *Marafiviruses* consistently reveals, as expected, the highest degree of disagreement. Interestingly, GSyV1 PRO has the least sequence similarity to any of the PRO domains of all *Marafivirus* endopeptidases analyzed and also shares the least similarity with TYMV (along with CSDaV) at 33.77%. These variations in sequence similarity can potentially be attributed to many factors from wide differences in hosts, host climate/ecosystem, and viral vectors.

### *Marafivirus* endopeptidases have auxiliary deubiquitinating activity

Importantly, the process of ubiquitination is reversible, allowing Ub molecules conjugated to various substrate proteins to be uncoupled after the cellular function(s) dictated by ubiquitination is complete (29). This reversibility of the Ub system is carried out by cellular DUBs, of which, there has been approximately 50 identified in *A. thaliana* alone (29, 31, 37, 42). Cellular DUBs are important in reversing Ub linkages to target proteins but also are essential in processing Ub precursor proteins (31, 42). Not surprisingly, viruses have acquired the ability to exploit the Ub system to their advantage by encoding for multifunctional proteolytic enzymes (often cysteine proteases) that not only assist with viral replication by processing the viral polyprotein but also acting as DUBs to shut down Ub-dependent host antiviral mechanisms (21, 43, 44, 45).

**Figure 2:**
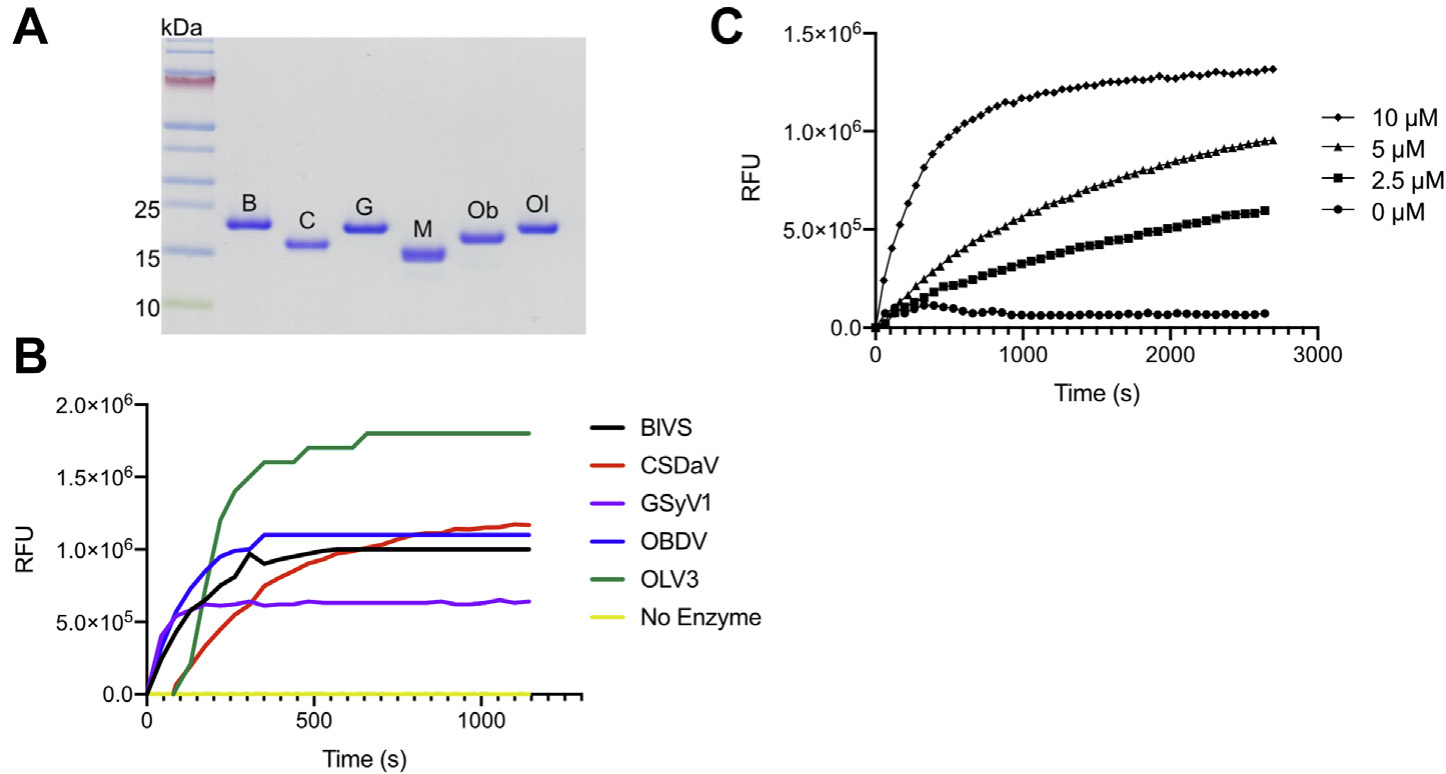
**Ubiquitin hydrolase activity of *Marafivirus* proteases.***A*, 10% TRIS Tricine SDS-PAGE gel of purified PRO domains of six *Marafiviruses* (blackberry virus S (B), citrus sudden death–associated virus (C), Grapevine Syrah virus 1 (G), maize rayado fino virus (M), oat blue dwarf virus (Ob), and olive latent virus 3 (Ol)). *B*, fluorescence *versus* time progress curve of *Marafivirus* proteases with Ub-AMC. Substrate was used at a final concentration of 200 nM and enzyme at 5 μM. Curves are colored with respect to enzyme (*black*–blackberry virus S (BlVS); *red*–citrus sudden death–associated virus (CSDaV); *purple*–Grapevine Syrah virus 1 (GSyV1); *blue*–oat blue dwarf virus (OBDV); *green*–Olive latent virus 3 (OLV3); *yellow*–substrate only). *C*, fluorescence *versus* time progress curve of MRFV PRO with Ub-AMC. The substrate was used at a final concentration of 200 nM, and the enzyme concentration was varied as indicated. Kinetic plots were designed in GraphPad Prism v.8.4.3. AMC, 7-amino-4-methylcoumarin; MRFV, maize rayado fino virus; PROs, proteases; Ub, ubiquitin.

When it was discovered that TYMV PRO had *bona fide* DUB activity, it was suggested that additional plant viruses may possess this function as well, including *Marafiviruses* (11, 19, 21). To assay for this potential DUB activity in *Marafiviruses*, we chose to study the enzymatic activity of six PRO enzymes from a range of *Marafivirus* species (Fig. 1*B*). Each PRO domain examined was based on the region known to exhibit endopeptidase and DUB activity from TMYV PRO and excluded the putative cleavage site at the putative PRO|HEL junction of each viral polyprotein as shown in Figure 1*B*. Each construct contained the conserved Cys and His residues (Fig. 1*B*, denoted with stars) that form the papain-like cysteine PRO active site, forming the catalytic dyad in which a cysteine nucleophile and histidine base work in concert to hydrolyze the scissile bond (29, 45). The recombinant PRO domains were stable *in vitro*, and each could be purified to homogeneity (Fig. 2*A*). The fluorogenic substrate Ub-AMC was used to assess the DUB activity for each enzyme (46). The fluorogenic substrate on its own has a relatively low signal; however, hydrolysis by DUB enzymes liberates the AMC fluorophore from Ub, which dequenches the fluorophore and results in a measurable signal (46). As seen in Figure 2*A*, the PRO domains of BlVS, CSDaV, GSyV1, OBDV, and OLV3 (black, red, purple, blue, and green, respectively) were incubated with Ub–AMC at a constant concentration and fluorescence measurements were taken over a 20-min period at an ambient temperature. A clear increase in fluorescence was observed for all five of the viral enzymes over time compared with a control lacking enzyme, which showed no appreciable Ub–AMC hydrolysis. These data confirm that *Marafivirus* PRO enzymes do exhibit DUB activity, further expanding the number of known viruses known to encode this activity. As all of the structural data we present here were for the *Marafivirus* type member PRO domain (MRFV PRO), a more thorough progress curve was generated to show enzyme concentration dependence (Fig. 2*B*). It can be seen that there is a clear concentration-dependent rate of increase in the fluorescent signal directly proportional to the relative amount of enzyme present per reaction. These findings confirm that *Marafivirus* PRO domains are also DUB enzymes.

**Figure 3:**
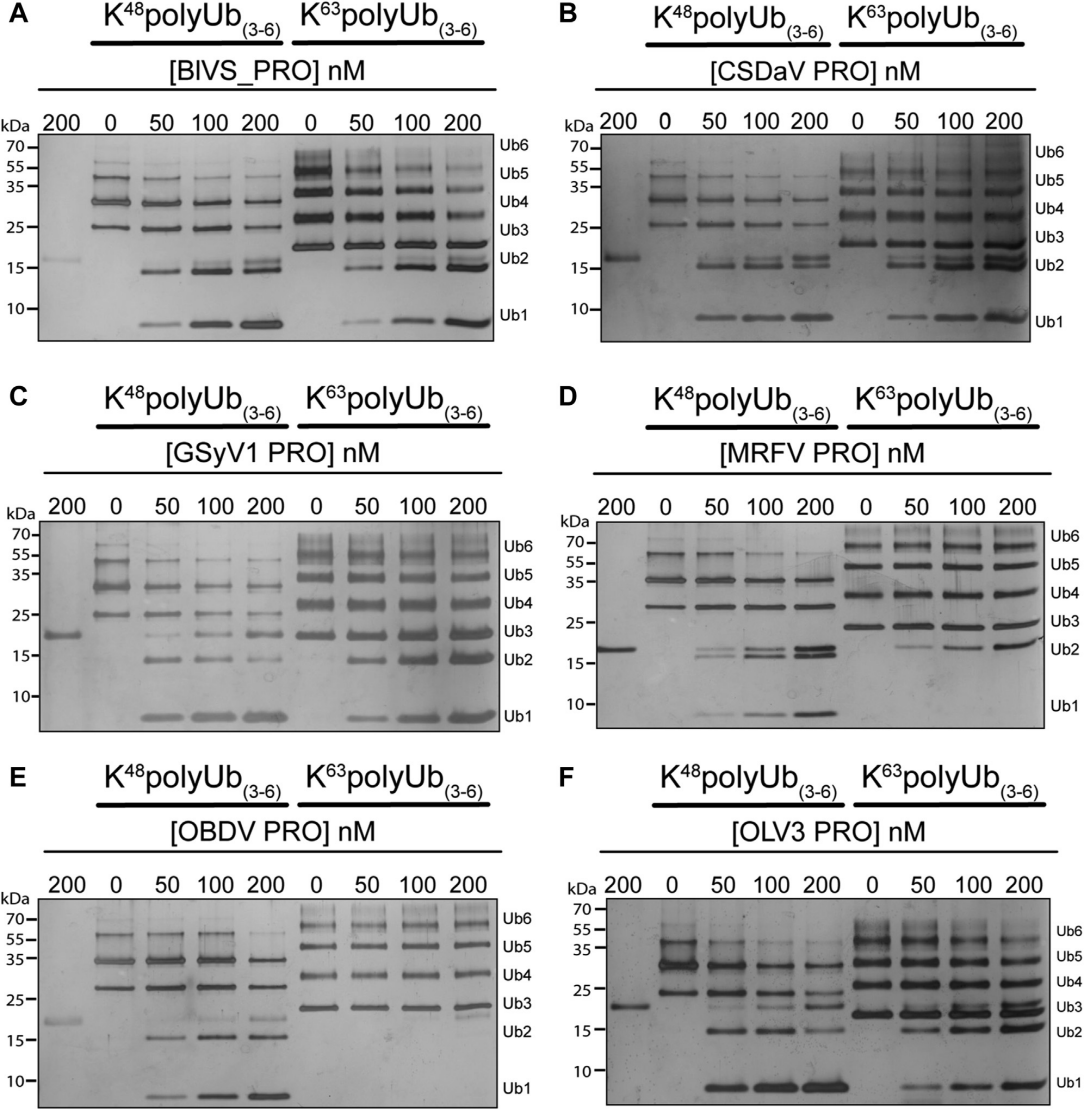
**Qualitative analysis of poly-Ub chain hydrolysis by *Marafivirus* proteases.***A*–*F*, K^48^/K^63^ poly-Ub chains in the presence or absence of variable concentrations of the indicated *Marafivirus* proteases/DUBs. Lane 1 for each assay contains protease alone as a reference. Ub chain lengths after hydrolysis are indicated. DUBs, deubiquitinases; Ub, ubiquitin.

As mentioned above, the cellular outcome of ubiquitination is often dictated by the topology of the Ub scaffold tethered to the target protein in the form of poly-Ub chains (29). To evaluate the substrate recognition ability of these PROs toward poly-Ub chains, each enzyme was coincubated with either K^48^/K^63^ poly-Ub chains (3–6 Ub molecules in length) to determine their substrate specificity. Upon mixing enzyme with each Ub chain type, the presence of di-/mono-Ub should accumulate, and higher molecular weight species diminish if Ub chain hydrolysis is occurring. As seen in Figure 3, *A*–*C* and *F*, BlVS, CSDaV, GSyV1, and OLV3 PRO all appear to hydrolyze both K^48^/K^63^ poly-Ub chains. A clear accumulation of di-/mono-Ub is present for both substrate types and increases with higher enzyme concentration. Interestingly, MRFV and OBDV PRO (Fig. 3, *D* and *E*) only appear to act on K^48^ poly-Ub chains. When comparisons are drawn between the PROs at the sequence level, MRFV PRO is more similar in sequence to BlVS and OLV3 PRO (∼49 and 55%, respectively—Fig. 1*C*) than in comparison with OBDV PRO (∼48%), whereas OBDV PRO is more similar to BlVS PRO (50%) and CSDaV PRO (64%); however, the preferences of MRFV and OBDV PRO toward Ub-substrate are shared. The preference of MRFV and OBDV PRO for K^48^ poly-Ub chains illustrates a surprising difference between these *Marafivirus* endopeptidases. It is possible that BlVS, CSDaV, GSyV1, and OLV3 PRO share a common structural feature that allows for broader substate specificity and is absent from MRFV and OBDV PRO. Unfortunately, there are too many differences between the enzymes at the primary sequence level to identify a region or motif that could be responsible for the difference in substrate specificity, and three-dimensional structural analysis would be needed to gain further insight.

By way of comparison, earlier studies illustrated that TYMV PRO deubiquitinates the RdRp of the TYMV replicase, rescuing the polymerase from proteasomal degradation *via* K48-polyubiquitination (21, 27). The DUB activity of TYMV PRO thus decreases RdRp turnover and appears to contribute to viral infectivity in plant cells (21, 27). Interestingly, it was also previously found that TYMV PRO is also able to hydrolyze K63 poly-Ub chains (21), but its intended target substrate in the cell remains unknown. The role of K63 uncoupling by viral plant DUBs is not well understood compared with mammalian system–affecting viral DUBs, which degrade K63 linkages to suppress innate immune signaling cascades (43, 45, 47, 48). We found that CSDaV, BlVS, GSyV1, and OLV3 also exhibit activity against K48 and K63-poly-Ub (Fig. 3), so it is possible that these enzymes uncouple Ub from a variety of cellular targets to promote viral replication, not just the viral RdRp. This is true for mammalian +RNA viruses that encode DUB enzymes (45), and additional studies may reveal this to also be the case for plant +RNA viruses. In contrast, we found that MRFV and OBDV PRO show a finer degree of substrate recognition than the other viral PRO enzymes, including TYMV PRO, showing activity only against K48 linkages. This suggests preference toward preventing RdRp degradation by the 26S proteosome or other aspects of K48 polyubiquitination in plants, such as plant development, hormone signaling, and cell cycle mediation (49).

### MRFV PRO facilitates polyprotein processing

Autocatalytic viral polyprotein processing by a PRO domain(s) encoded within the polyprotein represents a remarkably efficient mechanism of protein expression by +RNA viruses (22, 50, 51, 52, 53). Previous findings confirmed that TYMV’s polyprotein has at least two cleavage sites, which is carried out by the PRO domain (22, 40, 54). They are located between the PRO|HEL and HEL|RdRp junctions. We used MRFV PRO to gain insight into marafaviral polyprotein cleavage events and all subsequent experiments because this enzyme also proved amenable to structural studies and has considerable agricultural relevance. Furthermore, being the type member of the genus, we believe that it is most representative. To begin exploring the polyprotein processing by the MRFV PRO domain, two *E. coli* protein expression constructs were designed for the recombinant production of a subsection of the MRFV polyprotein spanning the PRO–HEL region. Two versions of the region were generated, one that contained a catalytically active PRO domain (WT) and another where the active site cysteine of PRO had been mutated (C61A). For both proteins, the PRO domain was expressed in its entirety, whereas only the N-terminal domain (ATP-binding domain) of the HEL was included. Importantly, however, the region contained the putative LVGA recognition site at the PRO|HEL junction (Fig. 1, *A* and *B*; cyan box). We predicted the site would be cleaved by the PRO domain although it contained a GA motif at the C-terminus, which is atypical for a DUB, which usually cleaves after a diglycine motif (GG). The complete PRO–HEL fusion would have been ∼60 kDa, while the truncated form is ∼44 kDa, which proved amenable for expression in *E. coli*. Cleavage at the predicted site by PRO would result in a ∼19-kDa N-terminal, His_10_-tagged PRO (with an enterokinase site in between the affinity tag and PRO), as well as an untagged version of the ATP-binding domain of the MRFV HEL (∼25 kDa) that would not be captured through affinity purification if cleavage by PRO were to occur.

**Figure 4:**
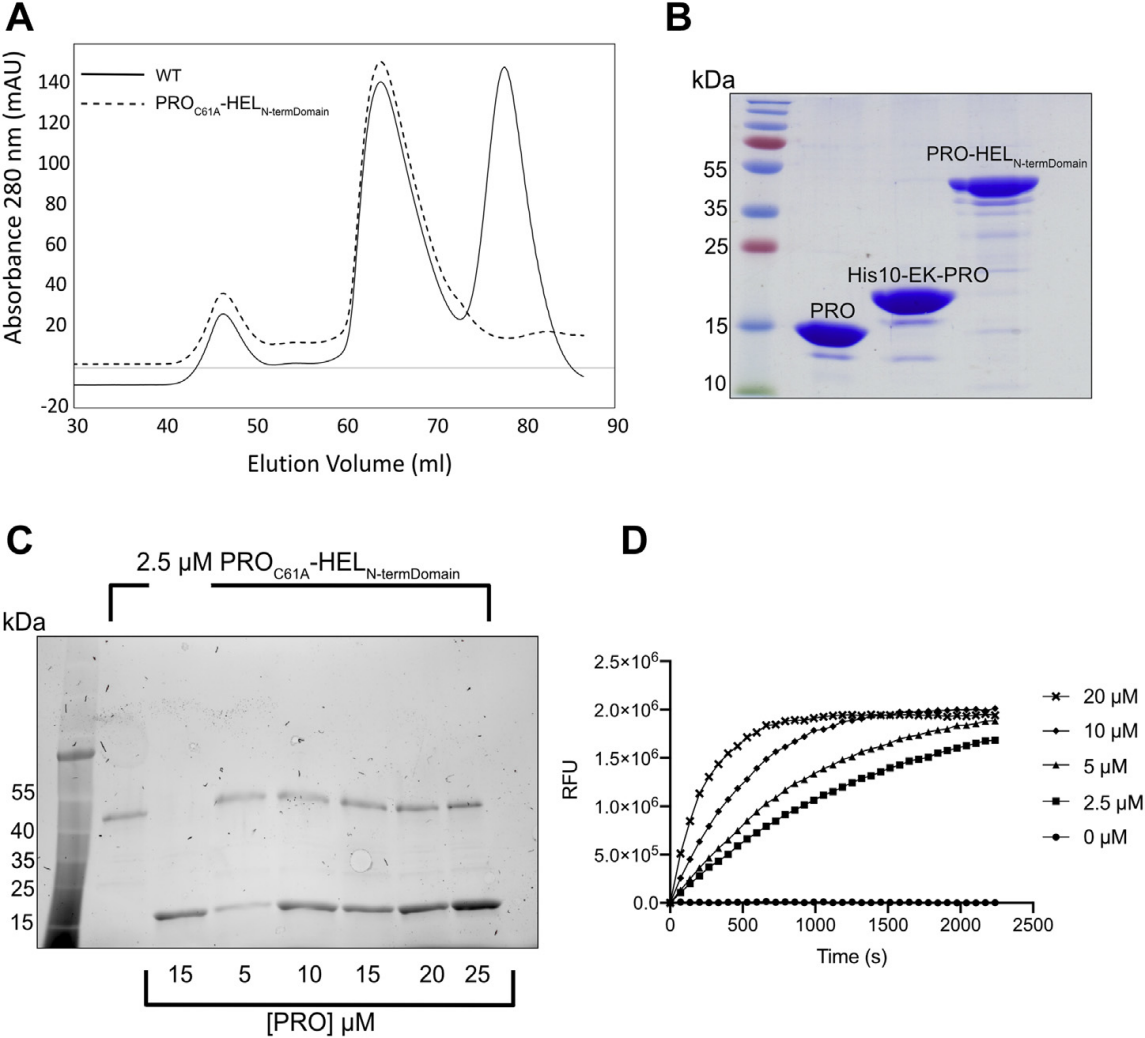
**MRFV PRO cleaves at the PRO–HEL junction in *cis* and recognizes a cleavage site reminiscent of the C terminus of ubiquitin.***A*, gel filtration chromatograms of the purification of MRFV PRO_C61A_–HEL_N-TermDomain_ and WT. *B*, 12% Coomassie-stained SDS PAGE gel of MRFV PRO from pGEX-6P-1 (∼16.4 kDa), in *cis*-cleaved MRFV PRO from WT MRFV PRO–HEL_N-TermDomain_ (∼19.4 kDa) and uncleaved WT MRFV PRO–HEL_N-TermDomain_ (∼44.1 kDa). *C*, 2.5 μM of purified MRFV PRO_C61A_-HEL_N-TermDomain_ was incubated with increasing concentrations of MRFV PRO for 30 min at 25 °C. Each reaction was subsequently loaded onto a 12% stain-free SDS-PAGE gel (Bio-Rad). *D*, fluorescence *versus* time progress curve of MRFV PRO with LRGG–AMC. The substrate was used at a concentration of 25 μM, and the enzyme concentration was varied as indicated. Kinetic plot were designed in GraphPad Prism v.8.4.3. AMC, 7-amino-4-methylcoumarin; HEL, helicase; MRFV, maize rayado fino virus; PROs, proteases.

Figure 4*A* depicts the gel filtration chromatograms of the WT and C61A PRO-HEL_N-TermDomain_ proteins. As shown for the active site mutant (dashed line), only one significant species is present at an elution volume of ∼65 ml, whereas in the WT trace (solid line), two species are present, suggesting autocatalytic cleavage of the PRO-HEL_N-TermDomain_ by PRO. For the WT protein, the larger species shares a nearly identical elution volume as the species containing the PRO active site mutant and likely represented the intact PRO-HEL_N-TermDomain_. Furthermore, for the WT protein, a second, lower molecular weight species appears at an elution volume of ∼80 ml. As stated above, cleavage at the proposed LVGA PRO|HEL junction would generate two additional proteins, only one of which (PRO) would be retained on a nickel affinity column as it is the only one that retains a His_10_ affinity tag. Figure 4*B* shows an SDS-PAGE gel of the species obtained from the WT gel filtration experiment. The intact full-length protein would have a theoretical molecular weight of ∼44 kDa, whereas the proteins generated by PRO-mediated cleavage would be ∼19 kDa (PRO domain) and ∼25 kDa (HEL ATPase domain). The processed HEL portion of the WT protein was lost in the purification process. Figure 4, *A* and *B* demonstrate that the WT PRO-HEL_N-TermDomain_ is processed at the predicted junction, however, not to completion.

To assess the nature of how PRO is acting to process the PRO|HEL junction, be it in *cis* and/or *trans*, a cleavage assay was carried out using the C61A mutant. As seen in Figure 4*C*, a constant concentration of the C61A mutant of PRO-HEL_N-TermDomain_ was incubated with an increasing concentration of active MRFV PRO domain. The PRO domain used in the experiment was identical to that used in the Ub hydrolase assays (Fig. 2*B*). The putative PRO cleavage site in the C61A mutant of PRO-HEL_N-TermDomain_ was not altered and thus remained cleavable. The results from Figure 4*C* indicate that even in a 10-fold molar excess of catalytically active PRO, the C61A mutant fusion protein was not processed in *trans* by the PRO domain; no species smaller than the full-length protein, excluding the added PRO domain (∼16 kDa), are visible. Should processing have occurred, both the larger PRO portion (∼19 kDa) of the PRO-HEL_N-TermDomain_ and ATP-binding domain of the HEL (∼25 kDa) would have appeared. Taken together, these results indicate that the *Marafivirus* PRO domain extracts itself from the viral polyprotein by *cis* cleavage of the PRO|HEL junction. This differs from previous observations in TYMV in which cleavage at the PRO|HEL junction appears to occur both in *cis* and *trans* (22). However, exclusive in *cis* cleavage is not uncommon in plant-affecting +ssRNA viruses, as is seen in members of the families *Potyviridae* and *Closteroviridae*, whose endopeptidases have been shown to also act solely in *cis* (55, 56). It could be that the release of PRO from the HEL domain enables PRO to adopt a fold that allows for in *trans* cleavage at the HEL|RdRp and RdRp|CP junctions, but this would require further studies.

As mentioned above, many PROs from +ssRNA viruses have auxiliary functions that aid in viral replication, including DUB activity. The C terminus of Ub is composed of the four amino acid motif LRGG. As demonstrated above and consistent with a number of other viral DUBs, *Marafivirus* DUBs can recognize the C terminus (LRGG) motif of Ub and cleave the bond downstream of the diglycine motif (29). Interestingly, the sequences at the PRO|HEL, HEL|RdRp, and RdRp|CP junctions all mimic the C terminus of Ub (Fig. 1*A*). Indeed, the predicted RdRp|CP junction in MRFV has an exact LRGG sequence and is very likely a PRO cleavage site. Although we did not carry out a cleavage assay as detailed as the PRO|HEL assay described above, Figure 4*D* illustrates the ability of MRFV PRO to hydrolyze the synthetic fluorogenic peptide LRGG-AMC at the scissile bond between the terminal Gly and AMC. A clear concentration-dependent trend is seen with increasing amounts of enzyme over time. In light of the X-ray structure of MRFV PRO described below, it is not surprising that LRGG-AMC is a poor substrate compared with Ub-AMC, as the peptide would have minimal interactions with the enzyme compared with Ub. Nevertheless, the LRGG-AMC assay indicates that *Marafivirus* PRO domains are able to recognize LRGG alone and very likely cleave at the RdRp|CP junction to liberate the major CP from the replicase proteins. Given our cleavage data, the HEL|RdRp junction is also most likely processed by PRO. The Ub-like LXG[G/A] sequence at the HEL|RdRp junction is conserved among the marafiviral polyproteins (Fig. 1*A*) and previous results of *Tymoviruses* processing at this junction have been demonstrated (40, 41, 54). Whether HEL|RdRp and RdRp|CP of the *Marafivirus* polyprotein are also processed exclusively in *cis* and the temporal nature of these events remain to be determined.

### Crystal structure of MRFV PRO

**Figure 5:**
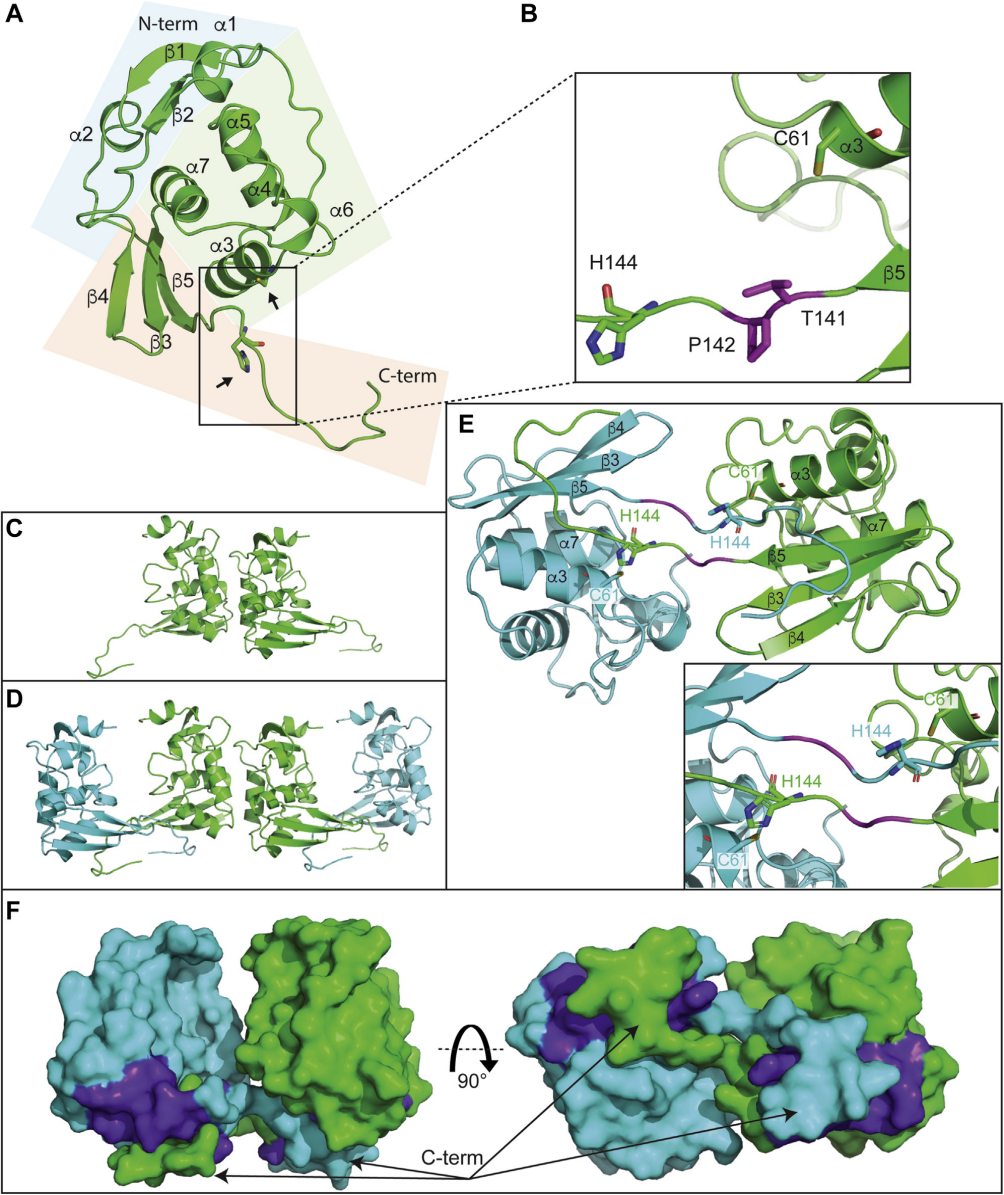
**Crystal structure of MRFV PRO.***A*, overall structure of MRFV PRO determined at 1.9 Å resolution. Individual domains of PRO are shown in *blocked colors* (domains 1, 2, and 3; *blue*, *green*, and *orange*, respectively). The catalytic residues are indicated with *arrows*. *B*, close-up of the active site architecture of MRFV PRO. *C*, asymmetric unit of MRFV PRO crystals. *D*, interaction of MRFV PRO symmetry mates. *E*, close-up on interaction of MRFV PRO symmetry mates, one of each is shown in *green* and *cyan*. Labeling is identical to that in panels *A* and *B*. *Inset* is a close-up of the symmetry mate active site residues. *F*, surface representation of MRFV PRO symmetry mates at two different angles. Figures were generated in PyMOL (71). MRFV, maize rayado fino virus; PROs, proteases.

We were able to crystallize MRFV PRO in space group P2_1_, and its three-dimensional structure was determined to a resolution of 1.9 Å using the TYMV PRO X-ray structure (PDB code: 4A5U) as an MR search model. PRO adopts a compact, three-domain fold (Fig. 5*A*), although its C-terminal conformation is held in place by a neighboring copy of the enzyme (Fig. 5, *D* and *E*). As seen in Figure 5*A*, the first domain (blue panel) is composed of a β-sheet (β1↑ β2↓) and ⍺-helices ⍺1 and ⍺2. The second domain (green panel) is predominantly a well-ordered five-helix bundle (⍺3-⍺7). Helix ⍺3 contains the catalytic cysteine nucleophile (C61) with its solvent-exposed thiol group at the N-terminal end of ⍺3 (Fig. 5*A* (arrows) and Fig. 5*B*). The third domain (orange panel) is comprised of a three-stranded β-sheet (β4↑ β3↓ β5↓) whose curved, open face packs against helices ⍺3 and ⍺7 of domain II. Domain III terminates with what appears to be a flexible loop that is solvent exposed until it inserts itself into the active site of a neighboring copy of the enzyme. This loop contains the active site histidine (H144) that is ∼17 Å from the catalytic cystine. Based on sequence alignments and a successful MR experiment, MRFV PRO is believed to have a simple Cys/His catalytic dyad similar to TYMV PRO (29).

The ∼17 Å distance between the side chains of C61 and H144 does not comprise a functional active site. The asymmetric unit (ASU) is composed of two copies of MRFV PRO (Fig. 5*C*) with most interactions occurring between the alpha helical bundles of domain II of each molecule. As seen in Figure 5*D*, the C-terminal tail of each copy of protein in the ASU traverses into its neighbor and appears to complete the active site of the neighboring molecule (Fig. 5*E*). The C-terminal tails of each symmetry mate jut into the cleft that exists between helices ⍺3 and ⍺7 as well as strand β5 of their neighbor (Fig. 5*E*). The catalytic C61 and incoming His144 residue of the neighboring symmetry mate arrange themselves to within a few angstroms of each other and appear coordinated for catalysis (inset to Fig. 5*E*). The active site arrangement of the crystallographic symmetry mates more closely resembles the structured active site of TYMV PRO (Fig. S1). Finally, surface representations (Fig. 5*F*) reveal the close association of the two MRFV PRO molecules that comprise Figure 5, *D* and *E*. The midsection of the C-terminal tails nestles itself into deep grooves formed between helices ⍺3 and ⍺7 of domain II, as well as strand β5 of the sole β-sheet of domain III. The ends of the C-terminal tails seamlessly pack up against the convex face of the β-sheet (purple). MRFV PRO is monomeric in solution according to size-exclusion chromatography (Fig. S2), and this dimeric interaction between the monomers of the ASU is most likely a crystallographic artifact.

### Comparing the PROs of MRFV and TYMV

**Figure 6:**
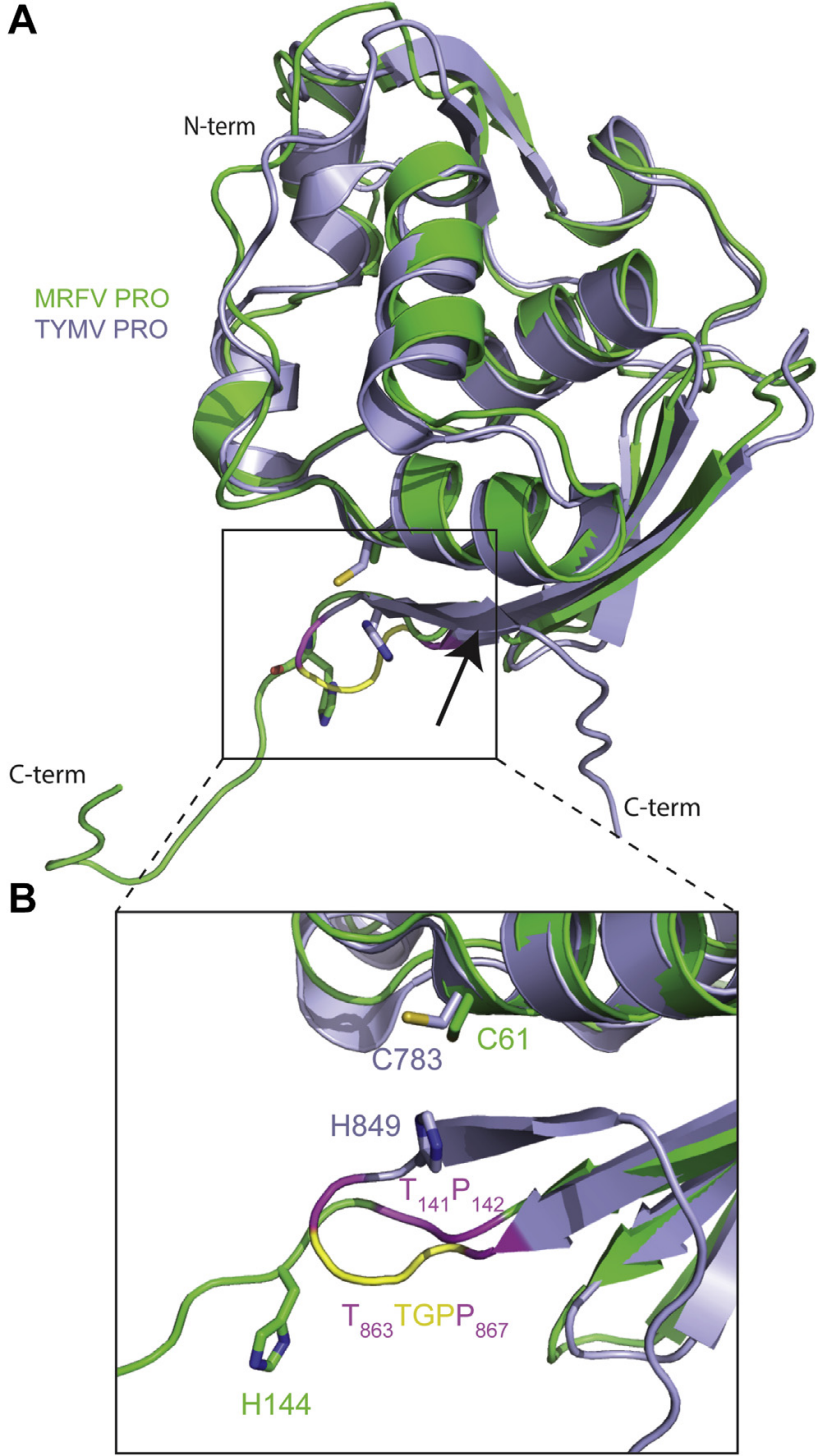
**Crystal structure of MRFV PRO superposed with TYMV PRO.** Overview (*A*) and close-up (*B*) of MRFV PRO overlayed with TYMV PRO (PDB code: 4A5U). MRFV PRO is shown in *green*, and TYMV PRO is shown in *pale purple*. T141 and P142 (MRFV) and the corresponding T867 and P867 (TYMV) are shown in *magenta*. The TYMV PRO loop composed of G^864^P^865^P^866^ is shown in *yellow*. Figures were generated in PyMOL (71). MRFV, maize rayado fino virus; PROs, proteases; TYMV, turnip yellow mosaic virus.

MRFV PRO and TYMV PRO share a similar three-dimensional fold (Fig. 6*A*) outside of variability in loops. Loops often play a large role in protein–protein interactions and substrate recognition (57), so it follows that different substrate/interaction requirements would manifest in loop variability between the MRFV and TYMV PROs. The MRFV PRO structure was compared with known three-dimensional protein structures using the DALI server (58), which revealed TYMV PRO to be the closest structural homologue, with a Z-score of 20.4 (PDB code: 4A5U). The next closest structural homologue was the ovarian tumor domain–containing protein 3 (OTUD3) from *Homo sapiens* with a Z-score of 5.9 (PDB code: 4BOU), followed by OTUD1 from *Saccharomyces cerevisiae* with a Z-score of 5.5 (PDB code: 3C0R). TYMV PRO itself has been characterized as a viral OTU DUB based on its overall core fold (24, 59) and appears to have more homology with OTUD3 and OTUD1 with Z-scores of 7.6 and 7.4, respectively. The difference in Z-scores of each PRO domain with OTUD1/3 can primarily be attributed to large variations in the C termini of MRFV and TYMV PRO. As mentioned previously, the C-terminal of the MRFV PRO structure differs compared with TYMV. For this reason, TYMV PRO has a more organized active site with its catalytic Cys783 and His849 residues in close coordinating distance, even in the absence of Ub substrate (Fig. 6*B*). Despite their differences, the structural homology of MRFV PRO shared with TYMV PRO, OTUD1, and OTUD3 clearly classifies it as a viral OTU DUB.

As seen in the sequence of TYMV PRO, there is a region at the C-terminal upstream of the catalytic His known as the “GPP flap” (outlined in black) (Fig. 1*B*). This motif has been shown to be essential for the PRO to toggle between endopeptidase and DUB activity (60). Mutations in the region decreased DUB activity but did not appear to hinder polyprotein processing at the PRO|HEL junction, indicating the importance of the flap to modulate Ub-dependent antiviral responses but not polyprotein processing (60). Interestingly, aside from the terminal proline (P142), our crystal structure of MRFV PRO reveals that the enzyme lacks a complete GPP motif (Figs. 1*B* and 6*B*). Glycine and proline are key residues in β-turns, and their absence may explain why MRFV PRO fails to form a four-stranded β-sheet following β5 as seen in the previously determined crystal structure of TYMV PRO (Fig. 6) (24). Interestingly, with the exception of MRFV PRO, all the *Marafivirus* PRO domains in our study appear to contain a loop region similar to the GPP loop of TYMV PRO, yet we found them to be fully capable of cleaving the viral polyprotein and poly-Ub chains. Indeed, as described below, we found that interactions of the enzyme with Ub prompts MRFV PRO to adopt a conformation that brings H144 close to C61 and generate a complete active site within a single monomer of MRFV PRO that would turnover Ub.

### The structure of MRFV PRO in complex with Ub

**Figure 7:**
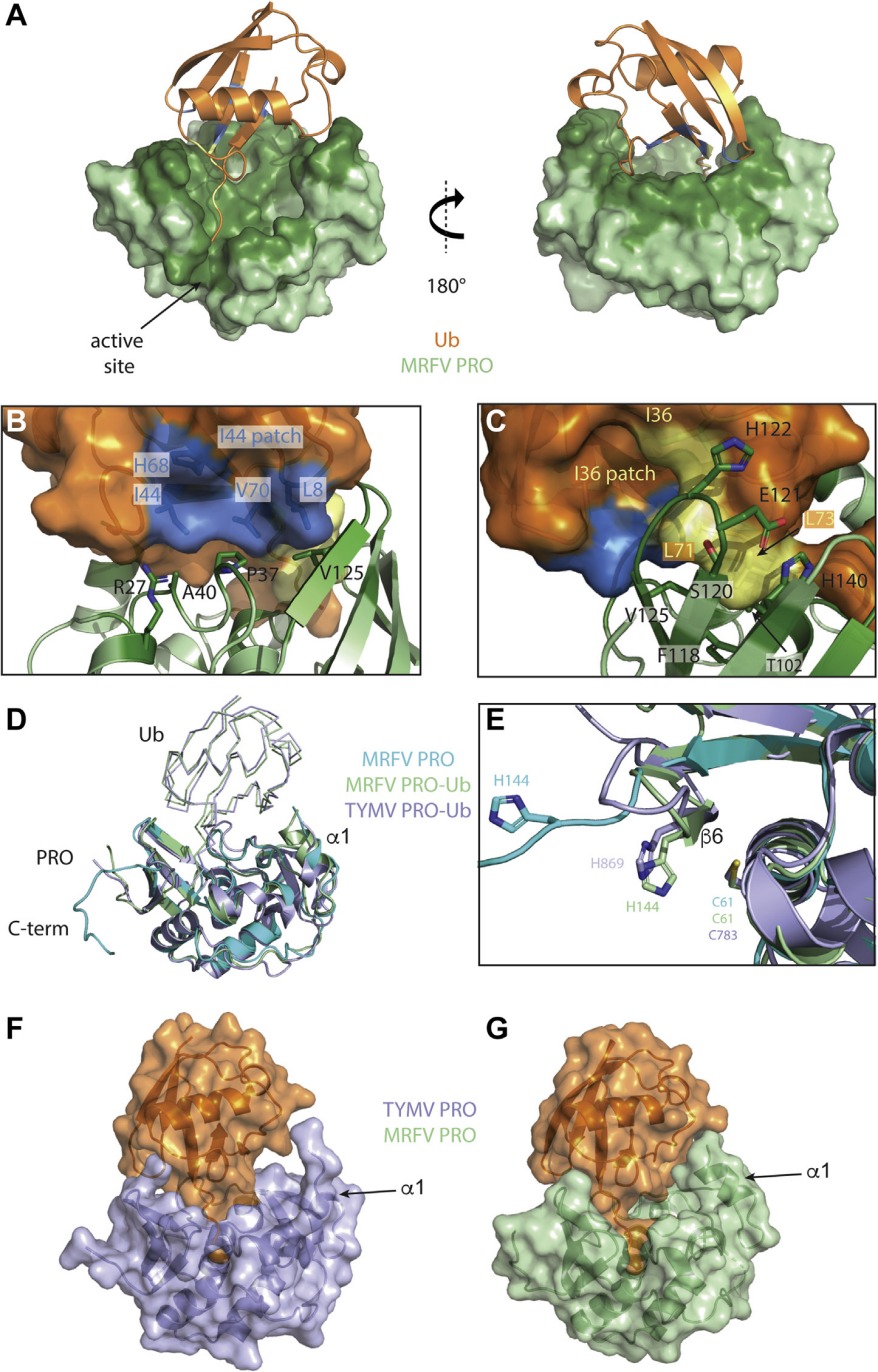
**Crystal structure of MRFV PRO bound to Ub and compared with TYMV PRO–Ub.***A*, MRFV PRO is shown as a surface representation and in *green* and Ub as a *cartoon* in *orange*. *Dark green* regions are interacting residues of PRO. *B*, interactions of the Ile44 patch of Ub (shown in *blue*) with key residues of PRO. *C*, interactions of the Ile36 patch of Ub (shown in *yellow*) with key residues of PRO. *D*, superposition of MRFV PRO, MRFV PRO–Ub, and TYMV PRO–Ub (PDB code: 6YPT). Ub molecules are shown in *ribbons*, and PRO domains are shown as *cartoons*. *E*, superposition of the active sites of MRFV PRO, MRFV PRO–Ub, and TYMV PRO–Ub. *F* and *G* surface representations of TYMV PRO–Ub and MRFV PRO–Ub. Figures were generated in PyMOL (71). MRFV, maize rayado fino virus; PROs, proteases; TYMV, turnip yellow mosaic virus; Ub, ubiquitin.

MRFV PRO was covalently linked to Ub. Specifically, Ub-3Br is a suicide substrate of deubiquitinating enzymes that is a modified form of WT Ub in that its C-terminus is modified to harbor a reactive C-terminal tail, which can irreversibly bind to the active site cysteines of DUBs through a covalent linkage (Fig. S3). The covalently linked protein complex was crystalized in space group I4. Figure 7*A* shows a remarkably large binding interface between MRFV PRO and Ub. The C-terminal tail of Ub nestles deep into the active site channel of PRO and numerous additional interactions of the enzyme with the beta-grasp fold of Ub (Fig. 7, *A* and *B*). As determined through the PISA server (61), there are 38 residues of PRO that are involved with interactions with Ub, which is over 25% of the residues, covering 948 Å^2^ (∼13%) of the accessible surface area.

Ub has four key surface features that are typically recognized by DUBs and Ub-binding proteins (28, 62). Two of these features are essential in how PRO recognizes Ub, which are the hydrophobic Ile44 and Ile36 patches (Fig. 7, *A*–*C*). The Ile44 patch is composed of residues L8, I44, H68, and V70. Figure 7*B* shows the interactions that occur at the Ile44 patch between PRO and Ub. Four key resides of PRO partake in the stabilizing of Ub on the surface of PRO. Hydrophobic residues P37 and A40 of the long loop connecting ⍺1 and ⍺2 grip against the hydrophobic Ile44 patch. The pyrrolidine side chain of P37 quite efficiently burrows into the groove in Ub created by I44 and V70. V125 of PRO also uses its hydrophobic isopropyl side chain to facilitate interactions with the lobe of Ub that arises from L8. Although not hydrophobic, R27 is a key residue for stabilizing Ub binding at this region because its guanidino group hydrogen bonds with Ub’s main-chain carbonyl group of G47 (Fig. S4*A*). This hydrogen bond orients the guanidino group to press up against the I44 patch and impart another degree of stabilization. Interestingly, only MRFV and OBDV have Arg residues at this position (Fig. 1*B*, triangle at position ∼30). R27, along with A45 (Fig. 1*B*, triangle at position ∼50), are the only two residues exclusively shared by OBDV and MRFV; however, A45 has no interactions with Ub. R27 is clearly important in MRFV PRO recognition of Ub and could be a factor that we can attribute into MRFV and OBDV lacking the ability to recognize and subsequently process Ub-K^63^ chains.

The Ile36 patch of Ub also has many interactions with PRO as seen in Figure 7*C*. V125, F118, and S120 are all directly involved with hydrophobic contacts with the Ile36 patch, helping to stabilize Ub on the surface of PRO while also facilitating entry and guidance of the C-terminal tail of Ub down into the channel that terminates with the active site. V125 is involved with hydrophobic interactions with both patches, showing its importance in recognizing Ub. Similarly, TYMV PRO has an Ile847 (highlighted by a triangle in Fig. 1*B*) at this position, and previous experimental findings have shown the importance of this residue in DUB activity (24). Finally, T102 of MRFV PRO forms two hydrogen bonds with L73 of Ub. The first is between the main-chain amide of T102 and the main-chain carbonyl of L73. The second is between the side-chain hydroxyl of T102 and the L73 main-chain amide (Fig. S4, *B* and *C*). H122 of PRO forms a hydrogen bond between N^δ1^ of its side chain and the side-chain amino group of Q40 of Ub (Fig. S4*D*). Finally, H140 of PRO forms a critical hydrogen bond between its side-chain N^ε2^ and the main-chain carbonyl of R74 of Ub (Fig. S4*E*). Although not all of these interactions are directly with Ile36 patch residues, they are key in this general vicinity and help stabilize Ub globally when considered together. Furthermore, the interactions of T102 and H140 interact directly through hydrogen bonding with the “LRGG” tail of Ub, suggesting that they also participate in polyprotein substrate recognition.

Overall, the crystal structures of MRFV PRO–Ub and TYMV PRO–Ub (PDB code: 6YPT) are similar (Fig. 7*D*). The core secondary structure and folds are maintained with only subtle variation within loop regions. Ub binds to both proteins in a similar orientation. Interestingly, in the presence of Ub, the C-terminal tail of MRFV PRO adopts a conformation highly similar to the GPP flap of TYMV PRO (unliganded and liganded) as there now is a turn following β5, which allows for the formation of a 4-stranded β-sheet and a more canonical, rigid active site (Fig. 7, *D* and *E*). Furthermore, MRFV PRO forms a much more extensive interaction with Ub than TYMV PRO, with a complex formation significance score (CSS) of 1.000 (scale being 0–1) as determined from the PISA server (61) (Fig. 7, *F* and *G*). TYMV PRO–Ub has a CSS of 0.822. Much of this is due to ⍺1 of MRFV PRO, which is more fully formed in the MRFV PRO–Ub structure than TYMV PRO–Ub and may also contribute to poly-Ub chain-type specificity. Interestingly, MRFV PRO (along with SARS-CoV-2 PLpro) have the highest CSS score of viral DUBs that have been structurally characterized bound to Ub, which includes Crimean–Congo hemorrhagic fever virus, equine arteritis virus, murine cytomegalovirus, Dugbe virus, Hazara orthonairovirus, mouse hepatitis virus, and Middle East respiratory and severe acute respiratory syndrome coronaviruses.

## Conclusion

Together, our results provide new structural and biochemical insights into the papain-like cysteine PROs present in the polyproteins of *Marafiviruses*. We demonstrate for the first time that these enzymes have deubiquitinating activity in addition to acting as endopeptidases that process the viral polyprotein. Polyprotein processing assays have provided first insights into how *Marafiviruses* process their polyproteins, which appear to have features distinct from their *Tymovirus* relatives. Our structural findings of the maize-affecting type member MRFV reveal that the enzyme has regions that appear to be highly dynamic, which assist in recognizing different viral and cellular substrates. Unexplored nuances exist that can still be investigated to understand how certain marafiviral DUBs selectively process only K48 poly-Ub chains and not K63 poly-Ub chains. Structural analysis also reveals that MRFV PRO has one of the most extensive interaction surfaces with Ub. Collectively, our results lay the groundwork in biochemically understanding this class of DUBs and sets the stage for future studies to exploit these enzymes.

## Experimental procedures

### DNA constructs

Synthetic DNA (Integrated DNA technologies) coding for ORFs of PRO domains from MRFV and citrus sudden death–associated virus (CSDaV) was amplified by PCR using primers listed in Table S1 and ligated into pGEX-6P-1 (GE Healthcare) using BamHI and XhoI restriction sites as shown in Table S1 (5′ and 3′, respectively). The remaining PRO ORFs from blackberry virus S (BlVS), Grapevine Syrah virus 1 (GSyV1), Olive latent virus 3 (OLV3), and oat blue dwarf virus (OBDV) were constructed by GenScript using the same cloning strategy.

The MRFV PRO_C61A_–HEL_N-termDomain_ fusion protein expression construct (residues 667–1038 from its polyprotein; UniProtKB-Q91TW9) was generated from codon-optimized synthetic DNA (Integrated DNA technologies) using primers FW 5′ GATATA**CATATG**CCGGAACCCGATACC 3′/REV 5′ TATATC**GGATCC**TTAGCAATAA AAGTCTACATAGG 3′ (NdeI and BamHI restriction sites shown in bold, respectively) and cloned into pET19b (Novagen) in frame with the native N-terminal His_10_ tag. The WT version of this protein (MRFV PRO-HEL_N-termDomain_), which retains the active site cysteine of PRO, was constructed using phosphorylated primers, and site-directed mutagenesis was used to reintroduce the cysteine that was formerly an alanine; FW 5′ P-CCGTGCCGCTTGCTTACTGGTCG 3′/REV 5′P-GTTGGATAAGGGATAGAG 3′ directly into the pET19b vector containing the insert.

### Protein expression and purification

Expression plasmids for the *Marafivirus* PRO domains were used to transform *Escherichia coli* BL21 (DE3) GOLD cells (Stratagene) for protein production. Transformed *E. coli* were grown overnight at 37 °C in LB containing 150 μg/ml ampicillin. The overnight culture was then used to inoculate 500 ml or 1 l of fresh ampicillin-containing LB (1:50 dilution) and was subsequently grown at 37 °C with shaking to an *A*_600_ of 0.7 to 0.8. Expression of the GST-tagged PRO enzymes (from pGEX-6P-1 constructs) or His_10_-tagged MRFV PRO_C61A_-HEL_N-termDomain_ was induced by the addition of 0.5 mM IPTG and left to incubate with shaking at 16 °C for an additional 18 h. Cells were then pelleted by centrifugation and either immediately used or stored at −80 °C.

All *Marafivirus* PRO domains were purified as follows. Cell pellets were resuspended in ice-cold lysis buffer (50 mM TRIS-HCl pH 8.0, 300 mM NaCl and 2 mM DTT) and lysed using an Avestin Emulsiflex C3 high-pressure cell homogenizer (ATA Scientific Instruments). Cell lysates were clarified by centrifugation (17,211*g* at 4 °C), and the supernatant containing GST-*Marafivirus* _PRO was mixed end-over-end for 1 h at 4 °C with GST-Bind resin (Millipore) that had been pre-equilibrated in the lysis buffer. The lysate/resin slurry was poured into a gravity column and washed with ∼20 column volumes of the lysis buffer, followed by elution of the fusion protein with the lysis buffer supplemented with 10 mM reduced glutathione (adjusted to pH 8.0 with NaOH). The GST tag was removed from each PRO domain using GST-tagged HRV 3C PreScission Protease (GE Healthcare), which was incubated with the eluted fusion protein in dialysis tubing overnight at 4 °C against 2 l of dialysis/gel filtration buffer (20 mM Tris HCl, pH 8.0, 150 mM NaCl, and 2 mM DTT). Tag-free PRO domains were separated from free GST and HRV 3C PreScission Protease using a Superdex 75 (GE Healthcare) gel filtration column. The concentration of each purified *Marafivirus* PRO was quantified using a NanoDrop (Thermo Fisher Scientific) instrument (*A*_280_, ε/1000 BlVS 11.46, CSDaV 16.96, GSyV1 13.98, MRFV 8.48, OBDV 9.97, OLV3 13.98 M^−1^ cm^−1^).

MRFV_PRO_C61A_-HEL_N-termDomain_ and the WT version–containing cell pellets were resuspended in ice-cold lysis buffer (50 mM Tris HCl, pH 8.0, 300 mM NaCl, 2 mM DTT, and 5 mM imidazole) and lysed identically to the PRO domains. Cell lysates were clarified by centrifugation (17,211*g* at 4 °C), and supernatants containing either protein were mixed end-over-end for 1 h at 4 °C with nickel-nitrilotriacetic acid resin (Qiagen) that had been pre-equilibrated in the lysis buffer. The lysate/resin slurry was then poured into a gravity column and washed with ∼20 column volumes of the lysis buffer, followed by ten column volumes of the lysis buffer supplemented with 15 mM imidazole, followed by ten column volumes of the lysis buffer supplemented with 30 mM imidazole and finally eluted with the lysis buffer supplemented with 250 mM imidazole. The eluted proteins were dialyzed against 2 l of dialysis/gel filtration buffer (20 mM TRIS-HCl pH 8.0, 150 mM NaCl and 2 mM DTT) overnight at 4 °C and then further purified using a Superdex 75 gel filtration column. The concentrations of purified MRFV_PRO_C61A_-HEL_N-termDomain_ or WT were quantified using a NanoDrop instrument (*A*_280_, ε/1000 = 39.42 M^−1^ cm^−1^).

Ubiquitin(1–75)–3-bromopropylamine (Ub-3Br) was prepared and purified as previously described (47, 63) for covalent coupling to MRFV PRO. This is a version of human Ub (UniProt P62987) lacking the terminal Gly. Purified Ub-3Br was dialyzed in 20 mM Tris HCl, pH 8.0, 150 mM NaCl, and 2 mM DTT, quantified by a Bradford protein assay and coupled with MRFV PRO in a 2-fold molar excess at 4 °C for 16 h with the addition of Tris(2-carboxyethyl)phosphine hydrochloride to a final concentration of 5 mM. The resulting MRFV PRO–Ub complex was separated from excess Ub-3Br by gel filtration.

### Enzyme assays

All six purified *Marafivirus* PROs were assayed against the fluorogenic substrate analogue 7-amino-4-methylcoumarin (AMC)–Ub (Ub-AMC) (Boston Biochem) or the synthetic peptide analogue LRGG-AMC (GenScript). The latter substrate represents the C-terminal motif of Ub as well as the linker between the RdRp domain and major CP of the MRFV polyprotein. Reaction buffer for all assays consisted of 50 mM Tris-HCl (pH 8.0), 150 mM NaCl, and 2 mM DTT. Substrates in the reaction buffer was placed in a black, flat-bottom 96-well microplate (Corning Life Sciences), and the enzyme was added immediately before readings. The final reaction volume was 100 μl. Time-course kinetics assays were carried out using a SpectraMax iD5 microplate reader (Molecular Devices). The instrument’s monochromators were set to excitation of 345 nm and emission of 445 nm. The PMT gain was set to medium, and reads were taken every 9 to 11 s.

### Polyubiquitin chain hydrolysis assays

Two hundred nanograms of the substrate (K48- or K63-linked poly-ubiquitin chains [Ub_3_-Ub_6_; Boston Biochem]) was incubated with 50 to 200 nM of each *Marafivirus* DUB in a reaction buffer identical to the abovementioned kinetics assay. Each reaction was incubated for 30 min at 25 °C. Reactions were terminated with the addition of 2X SDS-PAGE loading buffer. Reactions were visualized by carrying out TRIS-Tricine PAGE (10%) and subsequent detection using a Pierce Silver Stain Kit (Thermo Fisher Scientific).

### Protein crystallization

MRFV PRO was crystalized using the vapor diffusion method at 15 mg/ml in a condition that contained 100 mM Bis-Tris propane (pH 7.5), 200 mM sodium acetate, and 20% PEG 3350. Crystals appeared after ∼30 days at 4 °C. Crystals of the MRFV PRO–Ub complex were also grown using the vapor diffusion method in 100 mM phosphate citrate buffer (pH 3.8), 200 mM lithium sulfate, and 25% PEG 1000, which appeared after 1 day at 4 °C. Immediately before X-ray data collection, single crystals of both PRO and Ub-bound PRO were swept through a cryoprotectant composed of the initial crystallization condition supplemented with 25 or 15% glycerol (PRO and Ub-bound PRO, respectively) and subsequently flash-cooled in liquid nitrogen.

### X-ray data collection and structure determination

X-ray diffraction data were collected in-house at 100 K using a Rigaku MicroMax HF X-ray generator and R-AXIS IV++ image plate detector. Data were indexed using XDS for MRFV PRO (64) and iMOSFLM for MRFV PRO–Ub (65). Scaling was done using Aimless (66) as a part of the CCP4 i2 program suite (67). For the unliganded MRFV PRO structure determination, molecular replacement (MR) was carried out using the crystal structure of TYMV PRO with its cocrystallized contaminant excluded (PDB code: 4A5U). MR was done using PHENIX.PHASER (68) and was followed by model building using PHENIX.AUTOBUILD (68). Iterative model building and refinement was done using COOT (69) and PHENIX.REFINE (68). Structure determination for MRFV PRO bound to Ub was carried out almost identically; however, a multicomponent MR search was carried out using the structures of the unliganded form of MRFV PRO (determined herein) and Ub (PDB code: 1UBQ). Crystallographic and refinement statistics are provided in Table 1.

**Table 1:** Crystallographic and refinement statistics for MRFV PRO and MRFV PRO–Ub structures

	MRFV PRO	MRFV PRO–Ub
X-ray source	Rigaku R-AXIS IV++	Rigaku R-AXIS IV++
Crystal geometry		
Space group	P2_1_	I4
Unit cell (Å)	*a* = 43.20 *b* = 73.26 *c* = 54.34; α = β = γ = 90°	*a* = *b* = 75.72 *c* = 79.56; α = β = γ = 90°
Crystallographic data		
Wavelength (Å)	1.5418	1.5418
Resolution range (Å)	30.90–1.90 (1.97–1.90)^a^	27.42–2.09 (2.15–2.09)^a^
Total observations	89,460 (5639)	193,427 (15,741)
Unique reflections	27,471 (1718)	13,345 (1109)
Multiplicity	3.4 (3.3)	14.5 (14.2)
Completeness (%)	98.9 (99.6)	100 (100)
*R*_merge_	0.070 (0.41)	0.091 (0.36)
CC1/2	0.99 (0.74)	0.99 (0.97)
I/σI	7.03 (2.06)	26.3(8.0)
Wilson B-factor (Å^2^)	22.91	14.5
Refinement statistics		
Reflections in test set	2366 (235)	1296 (138)
Protein atoms	2175	1683
Ligands	0	16
Solvent molecules	169	239
*R*_work_/*R*_free_	0.20/0.24	0.20/0.26
RMSDs		
Bond lengths/angles (Å/°)	0.007/0.84	0.002/0.55
Ramachandran plot		
Favored/allowed (%)	97.23/2.42	97.65/1.41
Average B factor (Å^2^)	25.77	22.63
Macromolecules	25.34	21.76
Ligands	-	27.96
Solvent	31.38	28.42

a Values in parentheses refer to the highest resolution shell.

## Data availability

X-ray structures reported in this article are available in the Protein Data Bank (PDB) under PDB IDs 7MIA and 7MIC. All other data are presented in the article.

## Supporting information

This article contains supporting information.

## Conflict of interest

The authors declare that they have no conflicts of interest with the contents of this article.
