# Supplementary material for: The endopeptidase of the maize-affecting Marafivirus type member maize rayado fino virus doubles as a deubiquitinase
Source: J Biol Chem. 2021 Jul 12;297(2):100957. doi: 10.1016/j.jbc.2021.100957 (PMC8348309; doi:10.1016/j.jbc.2021.100957)
Supplement: Supplemental Figures S1–S4 and Table S1 [file mmc1.pdf]

The endopeptidase of the maize-affecting marafivirus type member Maize rayado fino virus doubles as a deubiquitinase

Ankoor Patel, Jessica McBride and Brian L. Mark*

Department of Microbiology, University of Manitoba, Winnipeg, Canada, R3T 2N2

* Corresponding author: Brian L. Mark

E-mail: brian.mark@umanitoba.ca

Supporting Information

Table S1. NCBI reference sequence identification numbers pertaining to complete genomes of all six marafivirus DUBs studied. Primers used for PCR amplification are presented for MRFV and CSDaV with restriction sites represented in bold.

**DUB/Protease**	**NCBI Reference Sequence**	**Primers (5' to 3')**
BlVS	NC_038328	(expression plasmid constructed by GenScript)
CSDaV	NC_006950	Forward<br>GATATA**GGATCC**TCCGATTGGGACCCCTCTC
		Reverse<br>TATATC**CTCGAG**TTAACGAGGGGTGGCGCTC
GSyV1	NC_012484	(expression plasmid constructed by GenScript)
MRFV	NC_002786	Forward<br>GATATA**GGATCC**CCGGAGCCTGACACGGCC
		Reverse<br>TATATC**CTCGAG**TTAAAGTGAGAAATTATCGGC
OLV3	NC_013920	(expression plasmid constructed by GenScript)
OBDV	NC_001793	(expression plasmid constructed by GenScript)

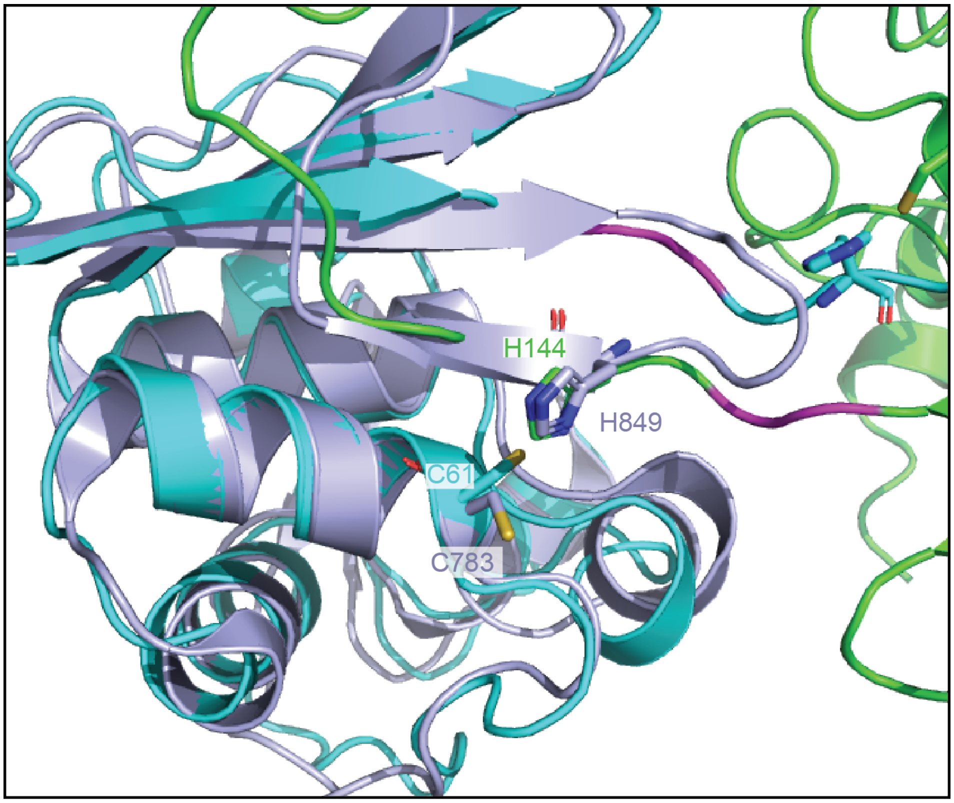

Figure S1. MRFV PRO symmetry mates aligned to TYMV PRO. MRFV PRO symmetry mates shown in cyan and green identically to Fig. 5 in main text. TYMV PRO is shown in lavender (PDB code: 4A5U). Active site residues are labeled.

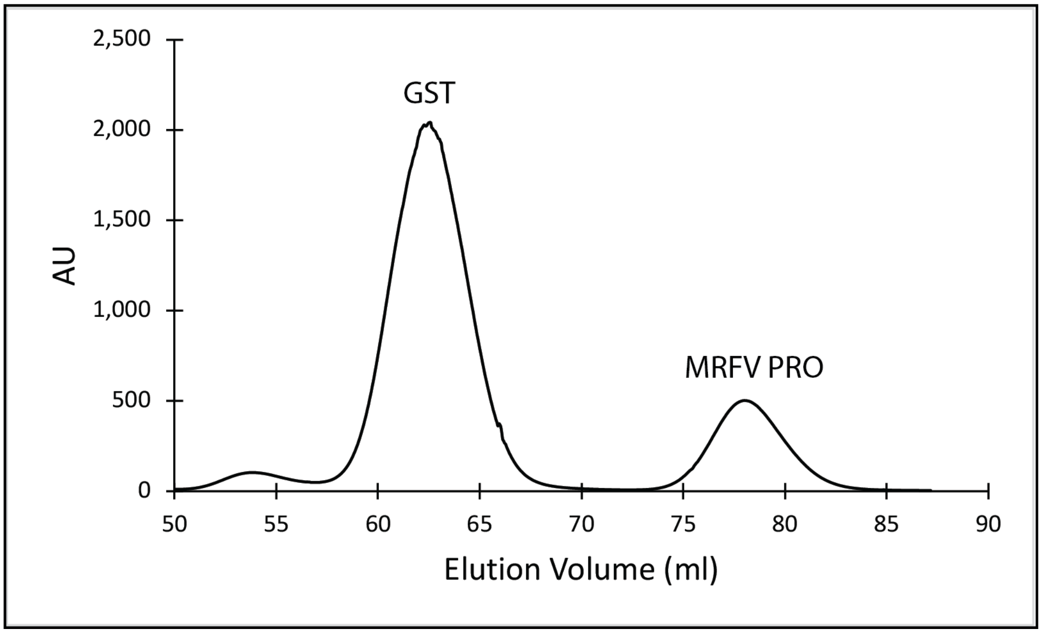

Figure S2. Size exclusion chromatogram of MRFV PRO.

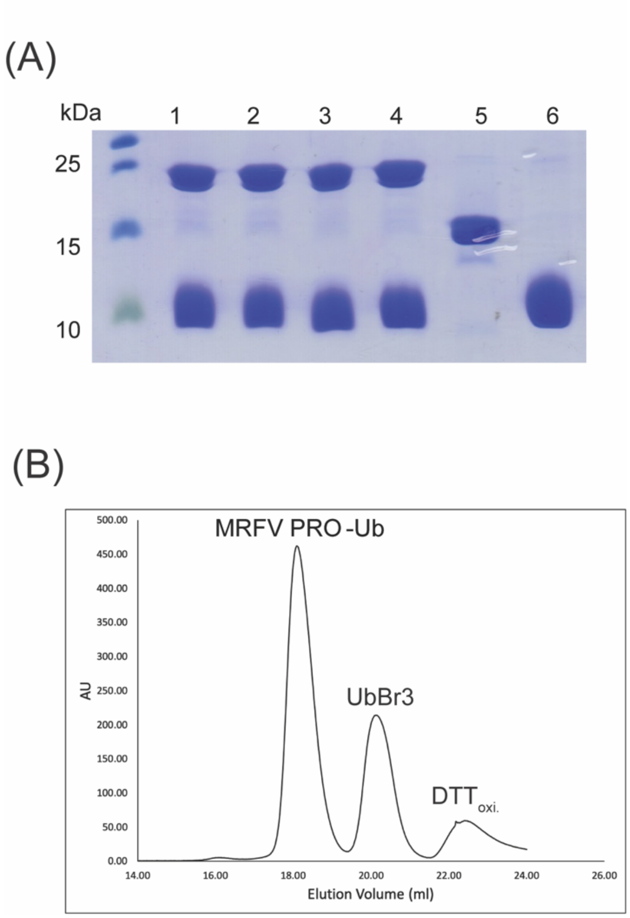

Figure S3. Formation and purification of the MRFV_PRO-Ub complex. (A) 15 % SDS-PAGE gel of test coupling reactions done with MRFV PRO and reactive UbBr3 (1:2 molar ratio) at various different temperatures for 1 h. 1 (4°C), 2 (16°C), 3 (25°C), 4 (37°C). Lanes 5 and 6 represent MRFV PRO (16 kDa) and Ub3Br (~9 kDa) alone. *(B)* Purifcation of the MRFV PRO-Ub complex by gel filtration using a Superdex 200 increase 10/300 (GE Healthcare). The first peak is indicative of the protein:protein complex, second peak is excess reactive UbBr3 and the final peak is oxidized dithiothreitol (DTT).

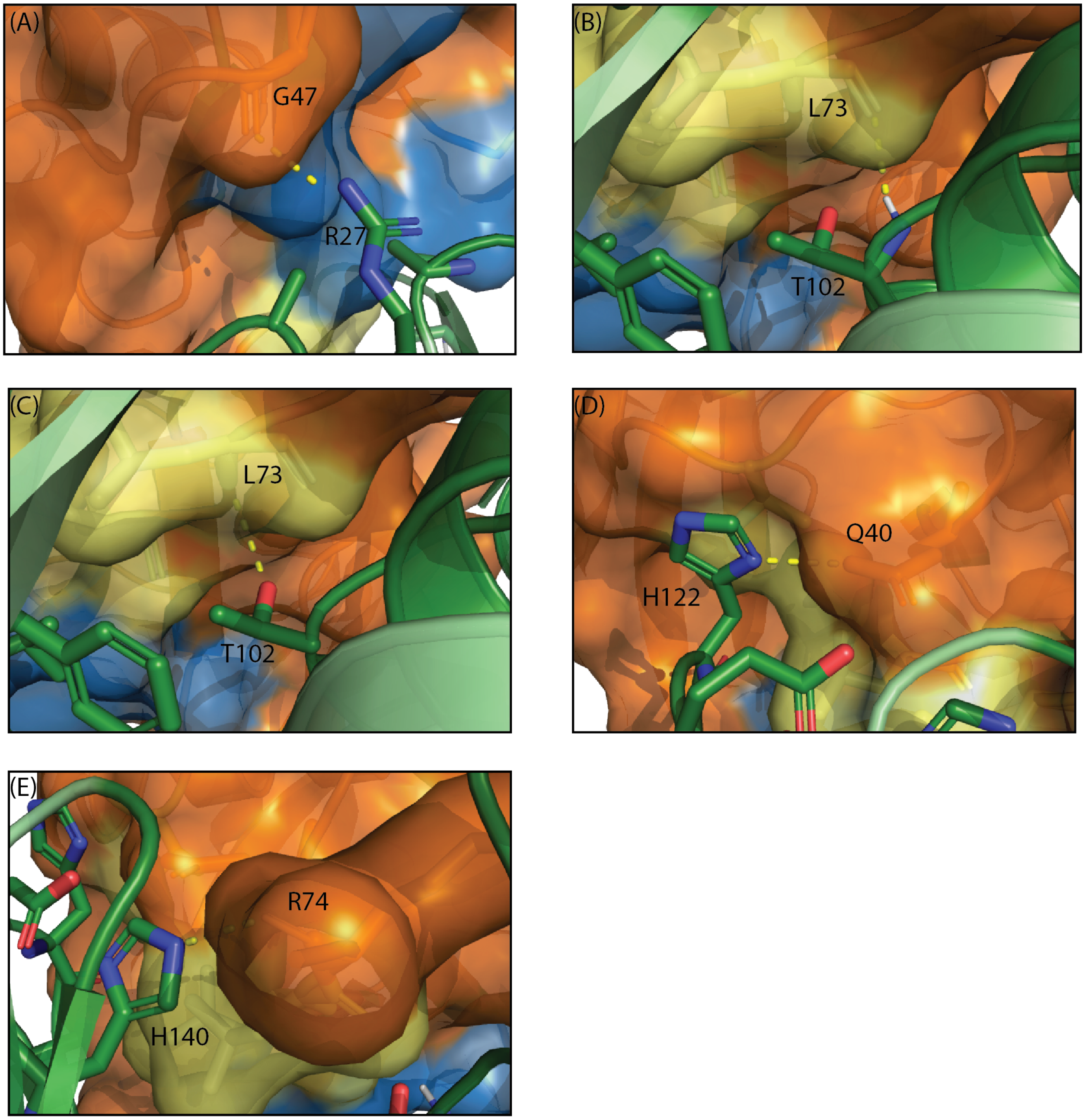

Figure S4. Hydrogen bonding interactions of MRFV PRO (green) and Ubiquitin (orange).
